# Seroprevalence and factors associated with exposure to *Neospora caninum* among dairy cattle smallholders in southern Rio Grande do Sul

**DOI:** 10.29374/2527-2179.bjvm004924

**Published:** 2024-12-24

**Authors:** Márcio Josué Costa Irala, Bianca Conrad Bohm, Ravena dos Santos Hage, Julia Somavilla Lignon, Fernando da Silva Bandeira, Fernanda de Rezende Pinto, Vinícius Silva Cheuiche Oberto, Robert Domingues, Alessandro Pelegrine Minho, Fábio Raphael Pascoti Bruhn

**Affiliations:** 1 Veterinarian, Departamento de Veterinária Preventiva, Universidade Federal de Pelotas, Pelotas, RS, Brazil; 2 Veterinarian, Centro Universitário da Região da Campanha – Urcamp, Bagé, RS, Brazil; 3 Veterinarian, Empresa Brasileira de Pesquisa Agropecuária – Embrapa, Bagé, RS, Brazil

**Keywords:** epidemiology, cattle raising, animal health, logistic regression, neosporosis, epidemiologia, pecuária, saúde animal, regressão logística, neosporose

## Abstract

*Neospora caninum* is a protozoan parasite that infects several species of animals (domestic and wild) and is one of the most common causes of abortion in cattle worldwide. To better understand the epidemiological chain of neosporosis, update the disease status and propose control measures to improve milk production in Rio Grande do Sul (RS), the present study aimed to evaluate the seroprevalence of *N. caninum* and its distribution in different municipalities of Rio Grande do Sul, Southern RS, Brazil, and determine the factors associated with exposure to *N. caninum* in small dairy cattle producers in this region. Cattle from 51 dairy farms located in nine municipalities in the southern region of RS were included in this study. Small dairy farmers were interviewed to collect information about the characteristics of their herds. The association between potential associated factors and seropositivity in cattle was assessed using a logistic regression model with a generalized estimating equation. Seroprevalence in individual animals and between herds was 33.9% (95% CI = 28.1–39.9) (121/309) and 80.4% (95% CI = 67.5–88.9) (41/51), respectively. The high seroprevalence found indicates that cattle are susceptible to exposure by *N. caninum* in a widespread manner in the Pelotas microregion. Furthermore, the sanitary management of cattle, the adaptation of tools that can improve the milk extraction technique and the technical monitoring of professionals who work on the properties can be effective alternatives for controlling *N. caninum.*

## Resumo

*Neospora caninum* é um protozoário parasita que infecta diversas espécies de animais (domésticos e selvagens) e é uma das causas mais comuns de aborto em bovinos no mundo. Com o objetivo de melhor compreender a cadeia epidemiológica da neosporose, atualizar o status da doença e propor medidas de controle para melhorar a produção de leite no Rio Grande do Sul (RS), o presente estudo teve como objetivo avaliar a soroprevalência de *N. caninum* e sua distribuição em diferentes municípios do Rio Grande do Sul, Sul do RS, Brasil, e determinar os fatores associados à exposição a *N. caninum* em pequenos produtores de gado leiteiro dessa região. Foram incluídos neste estudo bovinos de 51 fazendas leiteiras localizadas em nove municípios da região sul do RS. Pequenos produtores de leite foram entrevistados para coletar informações sobre as características de seus rebanhos. A associação entre potenciais fatores associados e soropositividade em bovinos foi avaliada por meio de um modelo de regressão logística com equação de estimativa generalizada. A soroprevalência em animais individuais e entre rebanhos foi de 33,9% (IC 95% = 28,1–39,9) (121/309) e 80,4% (IC 95% = 67,5–88,9) (41/51), respectivamente. A alta soroprevalência encontrada indica que os bovinos são suscetíveis à exposição por *N. caninum* de forma disseminada na microrregião de Pelotas. Além disso, o manejo sanitário dos bovinos, a adaptação de ferramentas que possam melhorar a técnica de extração do leite e o acompanhamento técnico dos profissionais que atuam nas propriedades podem ser alternativas eficazes para o controle de *N. caninum*.

## Introduction

Neosporosis, caused by *Neospora caninum*, is one of the main causes of reproductive disorders in cattle and has been identified as the primary reason of abortion in dairy herds globally, including Brazil (Schmidt et al., 2018). *N. caninum* has a wide host range (Khan et al., 2020). Canids (e.g., dogs, dingos, coyotes and wolves) are the definitive hosts of this protozoan, while many warm-blooded herbivores animals such as cattle, sheep, goats, horses, deer and even dogs, play the role of intermediate hosts (Cerqueira-Cézar et al., 2017; Khan et al., 2020). The intermediate hosts are infected through ingestion of water or food contaminated with the sporulated oocysts of *N. caninum*, which subsequently leads to the formation of tachyzoites and intracellular cysts in their tissues, mainly in the brain (Khan et al., 2020). Vertical (congenital) transmission of *N. caninum* in cattle is also an important route of infection (Fereig et al., 2022), which maintains the agent in herds for several generations, being considered the main route of infection in these animals (Andreotti et al., 2003; Khan et al., 2020). In this context, cows of any age can abort from three months to the end of pregnancy, with the majority of abortions occurring at five to six months. Fetuses may die in utero, be reabsorbed, mummified, autolyzed, stillborn, born alive with clinical signs, or born clinically normal but persistently infected (Dubey & Schares, 2011).

Neosporosis is a disease of high economic significance for global cattle farming, and it can lead to direct losses such as aborted fetuses and indirect losses such as veterinary assistance, diagnostic cost, and decrease in milk production (Monteiro, 2017). The study by Hernandez et al. (2001) demonstrated a 3 to 4% drop in milk production throughout the lactation period associated with exposure to *N. caninum*. In South America, neosporosis causes high losses estimated at approximately US$239.7 million/year. In Brazil, economic losses due to neosporosis are estimated to be approximately US$101 million/year and US$51.3 million/year in beef cattle farming and dairy farming, respectively (Reichel et al., 2013).

In recent years, the number of studies investigating the seroprevalence of bovine neosporosis in Brazil has increased; however, seroepidemiology has only been partially examined for some regions (Bruhn et al., 2013). In Brazil, the seroprevalence of anti-*N. caninum* antibodies in cattle varies from 6.7% to 97.2% (Cerqueira-Cézar et al., 2017). In Rio Grande do Sul (RS) in particular, last seroprevalence studies were conducted more than 10 years ago (Corbellini et al., 2002, 2006; Pegoraro et al., 2009; Ragozo et al., 2003; Vogel et al., 2006;), and the seroprevalence in the state ranged from 11.2% to 17.8%.

The RS is the third largest milk-producing state in Brazil. Therefore, considering the prominent role of RS in Brazilian dairy production and the importance of neosporosis as a hindrance to the milk production chain, the identification of links in the disease transmission chain and the determination of risk factors during milk production can enable to implement effective quality control measures in the environment that supplies the raw material (Hott et al., 2021).

To better understand the epidemiological chain of neosporosis in the RS region, update the disease status, and propose control measures to improve milk production in the state, the present study aimed to evaluate the seroprevalence of *N. caninum* and its distribution in different municipalities of the southern RS region, Brazil, and to determine the factors associated with *N. caninum* exposure in dairy cattle smallholders in this region.

## Material and methods

### Study area

**Figure 1:**
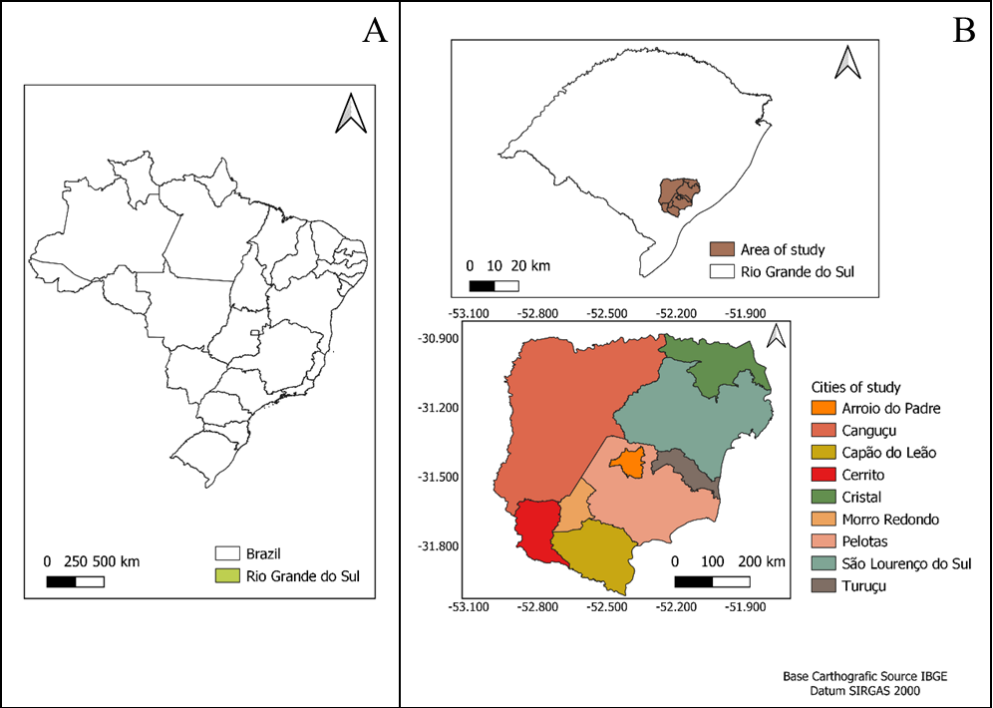
Map of the study area. (A) Map of Rio Grande do Sul with the location of the Pelotas microregion; (B) Map of the Pelotas microregion in greater magnification with the geographic points of the properties participating in the study (colored area).

A cross-sectional study was conducted to evaluate the association between the seroprevalence of *N. caninum* in dairy cattle and the possible factors related to *N. caninum* exposure. The seroprevalence of *N. caninum* in dairy cows was evaluated in 51 properties distributed among the municipalities of Cristal, São Lourenço do Sul, Canguçu, Turuçu, Morro Redondo, Arroio do Padre, Cerrito, Capão do Leão, and Pelotas (Figure 1). These cities constitute the Pelotas microregion, a milk-producing area located in southern RS, Brazil.

### Sampling and data collection

The herds were randomly chosen by the simple random sampling (draw) method from listings acquired from competent local authorities, such as the Empresa de Assistência Técnica e Extensão Rural (EMATER), Mixed Cooperativa Mista de Pequenos Agricultores da Região Sul Ltda (COOPAR), and the Cooperativa Sul-Rio Grandense de Laticínios Ltda (COSULATI).

The following inclusion criteria were considered: the presence of at least six cows in production in the area (in lactation) and an average production of at least 200 liter of milk/day. The initial approach was made through the authorized local people by explaining the research, its objectives, and its benefits and subsequently seeking their participation. The data collection period was April to October 2018.

The number of herds to be sampled was calculated using the Epitools Epidemiological Calculators (Sergeant, 2018) according to the estimated seroprevalence of *N. caninum* among animals and dairy herds located in the Pelotas microregion. Thus, based on the report of Bruhn et al. (2013), an exposure prevalence of 21% in animals and 95% in dairy herds was considered, with a margin of error of 5% and a confidence level of 95%, resulting in 255 animals and 51 herds to be sampled. On the basis of these values, approximately six animals per herd were sampled. For area selection, the COOPAR and COSULATI, which cover producers located in the southern region of the RS state, were consulted, as the herds were selected from a list of all producers of both cooperatives. Stratified random sampling (draw) according to the municipality was performed based on the drawing of the herds to be included in the sample. In the stratification process, approximately 40% of the herds linked to the cooperatives in each of the nine municipalities in the Pelotas microregion were sampled to complete the calculated minimum sample number of 51 properties.

To collect the required information, interviews were conducted using semi-structured questionnaires, which were previously tested to collect data regarding the possible factors associated with the occurrence of *N. caninum* and the perception of producers as decision-makers in managing the area. The questionnaires comprised 58 close-ended and 26 open-ended questions related to (1) area, (2) herd, (3) handling pregnant cows, (4) sanitary management, and (5) reproductive changes.

In addition to the questions on herd characteristics for each animal used for blood collection, the following details were obtained: abortion history (yes or no and the number of times of abortion), occurrence of any other reproductive complications (yes or no), and if yes, the type of complication. The interview was conducted by one interviewer, a veterinarian, with the decision-maker present in the area; data collection was performed previously through a pilot study involving participants to validate the questionnaire. The sampling period was approximately 8 months.

### Serology

For diagnosis, indirect ELISA was performed, which is a serological test recommended to evaluate the presence of *N. caninum* according to Wapenaar et al. (2007); this is a nonspecies-specific test and is advantageous for diagnosing neosporosis, as the protozoa has different host species. The indirect ELISA was performed according to the guidelines of the commercial kit for the *in vitro* diagnosis of *N. caninum* by the indirect immunoenzymatic assay (Imunodot Laboratory, Jaboticabal, SP, Brazil). The sera with a value above the cutoff limit of 5044 were considered positive at the percentage of 99.5% on each plate, according to the methodology described by Frey et al. (1998). A total of 309 bovine serum samples were processed in duplicate, and after performing the technique, the samples were read using an ELISA microwell plate reader device.

### Estimation of true seroprevalence

To calculate the true seroprevalence among herds, the sensitivity and specificity of the ELISA were adjusted from the individual to the herd level by Epitools Epidemiological Calculator (Sergeant, 2018), using the HERDACC (Jordan & McEwen, 1998) estimation method. Sensitivity (100%) and specificity (92%) values of the ELISA test were determined based on Blanco et al. (2014). Herd sensitivity and specificity were used to calculate the true seroprevalence at the herd level by using the Epitools Epidemiological Calculator (Sergeant, 2018).

### Statistical analysis

#### Geografic distribution

The distribution of *N. caninum* seroprevalence in each municipality in the Pelotas microregion was analyzed using thematic maps, which were constructed using the ArcGIS desktop 10.2.1 software. Shapefiles were used, which included a spatial data storage format consisting of the position, shape, and attributes of geographic characteristics, as reported by Instituto Brasileiro de Geografia e Estatística (2018). Maps were designed according to the cases of exposure superimposed on each municipality area.

Moran statistics (Anselin & Florax, 1995) were used to identify spatial clusters from the identification of high and low values of seroprevalence of *N. caninum* exposure in cattle. Global indicators of spatial autocorrelation (Moran I) provided a single measure for the set of all municipalities, thus characterizing the entire study region. The distribution patterns of the indicators were examined on a smaller scale by using the local Moran (LISA), which yielded a specific value for each municipality and enabled to visualize clusters of municipalities with similar values for the selected indicators (Melo & Mathias, 2010).

#### Univariate analysis

Databases were constructed from the data collected in the field by using Epidata 3.1 program. Subsequently, these databases were exported to SPSS 20.0 for statistical analyses.

Table 1 shows the main questions in the questionnaire form. To trace the profile of producers and milk production modes of producers located in the southern RS region, a descriptive analysis of the main variables was performed through the interviews.

**Table 1:** Main variables questioned in interviews conducted in 51 dairy farms in the Pelotas microregion, Rio Grande do Sul, Brazil.

**Item**	**Variables**
1. Property Features	Type of labor, level of education of workers who deal with cattle, main activity on the property, how long they have been in the dairy activity, total area of the property, and area for dairy cattle.
2. Herd Features	Lactation cows, dry cows, heifers, calves less than one year old, total number of herds, rearing of other animals on the property, amount of milk produced for industry and for consumption on the property, in addition to the system for raising cattle.
3. Management of the pregnant cow	Type of pre-partum maternity, condition of the cows at the time of calving, calving difficulty, and supplements used during pregnancy.
4. Reproductive changes	Occurrence of abortions and other problems such as birth of weak animals, retention of the placenta, repetition of heat, appearance of aborted fetuses, fate of the placenta, and handling performed with the cows that abort.
5. Handling sanitary	Type of food supplied to cattle, type of silo and silage used, form of food supply, vaccines, veterinary assistance, reproductive management, replacement of animals on the property, rotation of pickets, participation of animals in events, disposal criteria, use of quarantine, diseases, collection of carcasses, access of dogs in the cattle breeding area, presence of wild canids, food and way of raising the dogs.

For inductive statistics, as most of the herds showed at least one *N. caninum*-positive animal, the herds were categorized according to the seroprevalence. Thus, to enable the comparison of quantitative variables at the area level between the two categorized serological profiles, the herds were categorized based on the percentage of cattle exposure to *N. caninum*: up to 20% and more than 20% of seropositive cows. This categorization was based on the mean seroprevalence value found in the present study.

#### Multivariate analysis

To identify the factors associated with *N. caninum* exposure, initially, the dependent variable was transformed into a dichotomous one (animal or property: 0 - negative; 1 - positive), considering that the presence of a positive animal in the herd characterizes the property as positive. Subsequently, univariate analysis was performed using the chi-square test. Variables showing an association with a p-value of <0.02 in the chi-square test were selected for constructing the multiple binary logistic regression model.

#### Logistic regression

To identify the factors associated with *N. caninum* exposure among the animals, statistical analysis was performed using the serological results obtained from the ELISA test as a dependent variable and the animal-level variables (relative to each animal) collected in the interviews as independent variables, such as those related to the history of abortion occurrence and other reproductive complications in each animal.

The magnitude of the association was calculated using the adjusted odds ratio and its 95% confidence interval for the variable that showed a significant association (p < 0.05) in the logistic regression analysis.

The existence of an association between the possible risk factors at the herd level (collected through interviews) and seroprevalence was confirmed using the multiple generalized estimating equation (GEE) logistic regression model (Corbellini et al., 2006). Effects of variables on aspects were not discussed in this work; moreover, possible interactions between them were also not tested (Corbellini et al., 2006). For all variables present in the final model (p < 0.05), the risk was calculated using the adjusted odds ratio and its 95% confidence interval.

## Results

### Profile of dairy properties as perceived by producers

The median milk production on the properties was 250 liters (interquartile range [IR] = 550), with an area of ​​23 ha reserved exclusively for dairy production (animal facilities and resting and grazing areas; IR = 32). Most of the properties (80.4%) exclusively had family members in their workforce, and the educational level of the workers who managed cattle was minimal, that is, up to the 4th grade of elementary school (54.0%), with dairy farming as their main income activity (92.2%). Table 2 and 3 shows other variables related to the profile of the studied dairy properties.

**Table 2:** Variables related to productive characteristics among 51 dairy properties with lower (<20%) or higher (>20%)^1^ frequency of seropositivity for *N. caninum* (indirect ELISA), Pelotas microregion, Rio Grande do Sul, Brazil.

**Variable**	**General**	**<20%**	**>20%**
Number of residents	4 (3)	4 (3)	4.5 (2.7)
How long have you been in dairy farming?	20 (26)	30 (22)	15 (23)
Total property area	40 (57)	40 (51)	37 (61.7)
Area used for dairy cattle	23 (32)	25 (32)	21 (35.7)
Number of lactating cows	20 (26)	21 (22)	18,5 (26)
Dry cow herd	5 (7)	5 (7)	7 (11)
Heifer herd	9 (16)	6 (16)	10 (17)
Calf herd aged <1 year	8 (11)	7 (7)	10 (13,2)
Total herd strength	44 (63)	38 (63)	46.5 (64)
Production sold daily/milk	250 (550)	250 (590)	250 (448.7)
Production intended for breeding calves	10 (16)	10 (14)	8 (16)
Production intended for human consumption	2 (3)	2 (2)	2 (2.7)

1 The cutoff point of 20% was based on the average seroprevalence of animals on the evaluated farms.

**Table 3:** Variables related to property characteristics among 51 dairy farms evaluated with a lower (<20%) or higher (>20%)^1^ frequency of seropositive cows for *N. caninum* (indirect ELISA), Pelotas microregion, Rio Grande do Sul, Brazil.

**Variable**	**General (%)**	**<20%**	**>20%**
Type of labor			
Exclusively family	80.4	82.6	78.6
Fixed contractor	23.5	21.7	25.0
Temporary contractor	3.9	8.7	0.0
Mixed	2.0	4.3	0.0
Education level			
Incomplete elementary education	54.0	65.2	44.4
Complete primary education	6.0	0.0	11.1
Incomplete high school	4.0	4.3	3.7
Complete high school	20.0	17.4	22.2
Complete higher education	14.0	13.0	14.8
Main activity of the property			
Milk production	92.2	87.0	96.4
Agriculture	7.8	13.0	3.6
Wetlands or marshy areas			
No	54.9	52.2	57.1
Yes	45.1	47.8	42.9
Type of milking			
Channeled mechanics	45.1	39.1	48.1
Foot mechanics	54.9	60.9	51.9

1 The cutoff point of 20% was based on the average seroprevalence of animals on the evaluated farms.

According to producers, diseases (82.4%) were diagnosed in the majority of herds, mainly cases of bovine parasitic disease and clinical mastitis. More than half of the total properties do not have veterinary assistance (60.8%). The carcasses of dead animals or aborted fetuses were collected from the field (72.2%) and immediately buried (56.9%) in a location distant from the cattle herd facilities (Table 4).

**Table 4:** Variables related to sanitary management among 51 dairy properties with lower (<20%) or higher (>20%)^1^ frequency of seropositivity for *N. caninum* (indirect ELISA), Pelotas microregion, Rio Grande do Sul, Brazil.

**Variable**	**General (%)**	**<20%**	**>20%**
Diseases have been diagnosed in the herd in the last two years			
No	17.6	8.7	25.0
Yes	82.4	91.3	75.0
The property has veterinary assistance			
No	60.8	56.5	64.3
Yes	39.2	43.5	35.7
Carcasses of dead adult animals or fetuses are collected from the field			
No	27.1	33.3	22.2
Yes	72.9	66.7	77.8
Fate of dead animal carcasses			
Left in the field	23.5	21.7	25.0
Burned	7.8	8.7	7.1
Buried	56.9	52.2	60.7
Rodent control measures			
No	8.0	4.3	11.1
Yes	92.0	95.7	88.9
Presence of rats in the cattle feeding area			
No	21.6	17.4	25.0
Yes	78.4	82.6	75.0

1 The cutoff point of 20% was based on the average seroprevalence of animals on the evaluated farms.

### Seroprevalence and geographical distribution

Of the 309 bovine serum samples, 33.9% (95% CI = 28.1–39.9) (121/309) showed anti-*N. caninum* antibodies. Of the total participating farms, at least one *N. caninum-*seropositive bovine was identified in 80.4% farms (95% CI = 67.5–88.9) (41/51).

**Figure 2:**
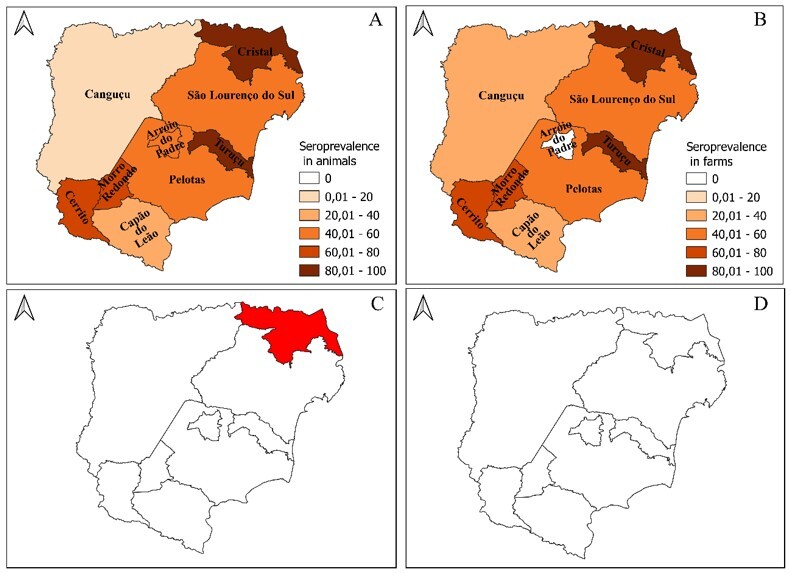
Seroprevalence distribution among animals by *N. caninum* in the microregion of Pelotas, 2018. (A) Descriptive distribution of seroprevalence rate among animals (positive animals/evaluated animals); (B) Descriptive distribution of seroprevalence rate among properties (positive properties/evaluated properties); (C) Cluster analysis estimated by Moran’s statistics, highlighting a clustering (in red) of the “high-high” type (p<0.05) of seroprevalence rate among animals (positive animals/evaluated animals); (D) Cluster analysis estimated by Moran’s statistics of the seroprevalence rate among properties (positive properties/evaluated properties) without significant clustering (p>0.05).

A higher prevalence of *N. caninum* occurrence was observed in the municipalities of Cristal, Turuçu, Cerrito, and Morro Redondo. A statistically significant cluster of seropositivity was noted among the animals (p < 0.05) reared in Cristal (Figure 2); this finding showed a higher exposure to *N. caninum* in cattle from this municipality than in cattle from other municipalities in the Pelotas microregion.

### Factors associated with N. caninum exposure

Univariate analyses indicated that the normal appearance of aborted fetuses (p = 0.028; OR = 7.07; 95% CI = 1.30–38.43), properties that purchase cows (p = 0.041; OR = 17.50; 95% CI = 1.22–250.35), and placenta left in open fields (p = 0.044; OR = 3.25; 95% CI = 1.10–10.39) were significantly associated with the seroprevalence of *N. caninum* in herds. Logistic regression analyses with the GEE (Table 5) indicated that the contact of dogs with cows (p = 0.003; OR = 5.24; 95% CI = 1.74–15.75) and properties that purchase cows (p = 0.018; OR = 5.46; 95% CI = 1.34–22.20) were more likely to have seropositive animals in the herd. In contrast, farms where dogs consumed raw bovine milk (p = 0.007; OR = 0.34; 95% CI = 0.15–0.74) showed a less likelihood of being seropositive for *N. caninum*.

**Table 5:** Results of multiple logistic regression analysis estimated by generalized estimation equation in relation to factors associated with *N. caninum* seroprevalence in dairy cows raised on 51 dairy properties in the Pelotas microregion, Rio Grande do Sul, Brazil.

**Factors**	**N**	**P-value**	**Odds Ratio**	**CI (95%)**
Air Silo/Pie				
No	144	0.435	-	-
Yes	153			
Intensive System			-	-
No	291	0.130		
Yes	18			
Low frequency of wild canines around the property				
No	249	0.959	-	-
Yes	60			
Dogs have contact with cattle				
No	12	0.003	1	
Yes	285		5.24	1.74-15.75
Placenta left in the open field				
No	128	0.316	-	-
Yes	181			
Properties that buy cows				
No	54	0.018	1	
Yes	38		5.46	1.34-22.20

## Discussion

The data on exposure to *N. caninum* in animals and farms in the present study revealed a higher seroprevalence of the parasite in the Pelotas microregion (33.9%) compared to the serological results reported in Pegoraro et al. (2009), over ten years ago, through the indirect immunofluorescence assay (IFA), where the seroprevalence rate in animals was 12.4% among 43 dairy herds in the same region. However, variations in serological techniques and differences in cutoff values used for seroprevalence analysis in seroepidemiological studies should be considered. Despite the different serological techniques used, both tests (ELISA and IFA) are diagnostic methods used in epidemiological studies, indicated to assess exposure and the risk of infection by this parasite in a herd (Andreotti et al., 2003). The seroprevalence observed in this study was also higher than that reported previously (11.2–17.8%) through IFA (Corbellini et al., 2002, 2006; Ragozo et al., 2003) and ELISA (Vogel et al., 2006) in other regions of RS. In contrast, the seroprevalence values detected among the evaluated animals were identical to those reported in other serological studies conducted in cattle herds in other Brazilian states using ELISA tests. For example, the seroprevalence reported in Amazonas and São Paulo was 30.2% (Azevedo Filho et al., 2021) and 35.5% (Sartor et al., 2005), respectively. Approximately similar values were reported in other countries, such as Argentina (35.3%) (Pereyra et al., 2021) and Colombia (28.3%) (Llano et al., 2018).

The main reason for the difference in seroprevalence in the studied region is that RS has witnessed climate changes over the last few years, where the temperature ranges between the different seasons have decreased. In addition, the relative humidity in the region has always been considered high. According to Dubey et al. (2007), mild temperature and humidity contribute to greater viability of oocysts eliminated in dog feces in the environment and favor faster sporulation, which can increase the probability of exposure by cattle. In addition, the protozoan is transmitted vertically (congenitally) in cattle, through several generations, which maintains the infection in herds, being considered the main route of infection in these animals (Andreotti et al., 2003).

The seroprevalence of *N. caninum* in the studied animals was mainly associated with the characteristics of sanitary management and reproductive changes. The purchase of cows was associated with a higher seropositivity in animals in the herd. Despite efforts to continue production, most properties showed a low rate of implementing advanced technology; for example, the lack of veterinary care observed in most rural establishments (60.8%) hindered decision-making based on technical criteria. Most farms purchase animals without performing serological testing. In other words, the lack of biosecurity increases the probability of introducing cattle into the herd that may be seropositive for *N. caninum*. Consequently, this increases the possibility of positive individuals being born (congenital transmission) and/or the occurrence of abortions. In this case, if dogs have access to placental and fetal remains, they can continue the parasite cycle, thus contributing to the spread of the disease within the properties. We also highlight that in RS (study location), it is common to use shepherd dogs in the management of animals on rural properties, and this proximity favors the *N. caninum* cycle.

Although we did not evaluate the infection in dogs, their presence in contact with cattle was associated with a increased seroprevalence between herds, as dogs are the main definitive host and excrete oocysts in their feces (Anvari et al., 2020; Appelt et al., 2019; Azevedo Filho et al., 2021; Venturoso et al., 2021). Macchi et al. (2020) showed evidence that the number of dogs was associated with high levels of infection, where the greater the number of dogs, the greater the risk of presenting high levels of infection. Considering the proximity between dogs and cattle reported on most properties, there is a high chance of dogs (96.1%) being infected by ingesting placental material from cattle and subsequently contaminating the rural environment with oocysts present in feces. According to Corbellini et al. (2006) dogs on smaller farms have greater and easier access to carcasses of aborted cattle, placentas and fetuses, favoring the horizontal transmission of *N. caninum*. Mello et al. (2008) suggested that dogs can also transport aborted fetuses and placentas from one location to another, which serves as food for wild canines and increases the likelihood of transmitting *N. caninum*. In contrast, Klun et al. (2019) found no association between the presence of dogs on farms and seropositivity of cows for *N. caninum*; Appelt et al. (2019) also did not observe seropositive dogs on farms where cows were tested positive for *N. caninum*. However, both authors indicated that the presence of dogs on farms can facilitate the spread of the protozoan parasite.

Currently, there are no commercially available vaccines to prevent neosporosis. Control strategies are limited to management techniques, such as serological testing in herd animals and those to be included in it, selective breeding based on seronegative animals, disposal of seropositive animals and their offspring, prevention of dogs' access to fetuses, placentas, and raw meat. Additionally, measures include avoiding contamination of cattle with oocysts eliminated in dog feces (e.g., reducing contact between cattle and dog feces, treating animals, reducing the number of dogs on properties, protecting the place of food and water supply, and removing feces) (Andreotti et al., 2003). According to Dubey et al. (2007), a general strategy to control neosporosis globally is not applicable because of regional differences in the epidemiology of bovine neosporosis; therefore, it is prudent to thoroughly study the regional epidemiology of neosporosis to implement control programs.

The present study has some limitations. First, the study used a cross-sectional design, which is useful to generate a hypothesis but fails to derive causal inferences; this is because animals were tested only once, which makes it difficult to establish causal associations when the samples and outcomes are not contemporaneous. Second, this study reflects the productive, sanitary, and cultural characteristics of cattle farming in the studied region, based on the producers' perception, as these informations was exclusively collected through interviews; consequently, the external validity of this type of study is limited. Third, considering that the seroprevalence of *N. caninum* exposure has increased in Brazil in recent decades and that we used an ELISA test validated long ago before its application as a diagnostic method, it is possible that an information bias was generated in the study because of changes in the predictive values of the diagnostic test with the expected increase in prevalence over recent years. Despite these limitations, the seropositivity observed in animals, associated with the frequency of the agent among dairy herds, indicates that *N. caninum* is widely disseminated in the Pelotas microregion and that cattle in this region may be more susceptible to high exposure to this protozoan. Additionally, the main associated factors observed in the present study indicate that the management of cattle healthcare, adequacy of tools that can improve the technique of milk extraction, and the technical monitoring of professionals working in the properties could be effective alternatives for controlling *N. caninum*.

## Conclusions

Our results show high seroprevalence in the animals and properties studied, indicating that cattle are susceptible to exposure by *N. caninum* in a widespread manner in the Pelotas microregion. They also demonstrate that the main associated factors observed in the present study indicate that the health management of cattle, the adequacy of tools that can improve the milk extraction technique and the technical monitoring of professionals who work on the properties can be effective alternatives for controlling *N. caninum*.
